# The HSP90/Akt pathway may mediate artemether‐induced apoptosis of Cal27 cells

**DOI:** 10.1002/2211-5463.12711

**Published:** 2019-08-30

**Authors:** Jianhua Wu, Lei Li, Yiting Wang, Xiaobin Ren, Ken Lin, Yongwen He

**Affiliations:** ^1^ Department of Periodontology The Affiliated Stomatological Hospital of Kunming Medical University Kunming China; ^2^ Department of Head and Neck Surgery The Third Affiliated Hospital of Kunming Medical University Kunming China; ^3^ Department of Oral and Maxillofacial Surgery The Affiliated Stomatological Hospital of Kunming Medical University Kunming China; ^4^ Department of Otolaryngology, Head and Neck Surgery Kunming Children's Hospital Kunming China; ^5^ Department of Dental Research The Affiliated Stomatological Hospital of Kunming Medical University Kunming China

**Keywords:** Akt, apoptosis, artemether, HSP90, tongue squamous cell carcinoma

## Abstract

Tongue squamous cell carcinoma is the most common malignant tumor in oral and maxillofacial regions. Recent research has found that artemether can inhibit growth and induce apoptosis of cancer cells, although the mechanism is not clear. The present study aimed to explore the correlation between the HSP90/Akt pathway and artemether‐induced apoptosis of Cal27 cells. A cell counting kit‐8 and flow cytometry were used to detect the proliferation and apoptosis of Cal27 cells, respectively, mRNA expression was examined by quantitative RT‐PCR, and protein expression was detected by western blotting. Our data revealed that artemether can inhibit growth and induce apoptosis of Cal27 cells. As the artemether concentration was increased, we observed downregulation of the expression of HSP90, p‐Akt and p‐mTOR in Cal27 cells, whereas the expression of Akt was not significantly changed. We also observed a time‐dependent decrease in the expression of HSP90, p‐Akt and p‐mTOR during exposure to 0.1 mg·mL^−1^ artemether. In conclusion, the HSP90/Akt pathway may be involved in artemether‐induced apoptosis of Cal27 cells.

**Abbreviations**
ANOVA: analysis of variance
CCK‐8: cell counting kit‐8
DMEM: Dulbecco's modified Eagle's medium
FITC: fluorescein isothiocyanate
HSP: heat shock protein; mTOR; mammalian target of rapamycin; PBS; phosphate‐buffered saline; PI; propidium iodide
TSCC: tongue squamous cell carcinoma
TUNEL: terminal deoxynucleotidyl transferase dUTP nick end labeling

Tongue squamous cell carcinoma (TSCC) is the most common malignant tumor in oral and maxillofacial area, characterized by high degree of malignancy, a high proliferation rate, strong invasiveness and easy occurrence of neck lymphatic metastasis 1, 2, 3. At present, chemotherapy is still one of the key methods to treat metastatic tumors. However, most chemosynthetic drugs have disadvantages, such as poor water solubility, low bioavailability, low selectivity and serious toxicity in the clinic 4. Thus, there is an urgent need to develop new anti‐tumor drugs for refractory and relapsed patients.

In recent years, botanical antineoplastic drugs have attracted much attention because of their unique physiological activity and low toxicity 5, 6. Many studies have shown that artemisinin, an antimalarial drug, and its derivatives have obvious cytotoxicity and inhibitory effects on tumor cells 7. Artemether, a methyl ether derivative of artemisinin, was also reported to inhibit the growth, invasion and migration of a variety of cancers, including stomach, liver and breast cancer 8, 9, 10, 11. These studies suggested that artemether is a potential anti‐tumor drug, although the specific mechanism remains to be clarified.

Heat shock proteins (HSPs) is a set of protective proteins. Under physiological stress or pathological conditions, the synthesis of HSPs in cells significantly increased, which has a great effect on the survival of organisms in severe environment. HSP90 receptor proteins are related to the apoptosis pathway, cell cycle and regulation of signal transduction, and can affect the growth or survival of cancer cells through its receptor by multiple links and pathways 12, 13.

Akt is a serine–threonine kinase tightly connected with signaling pathway induced by growth factor and has an important effect on cell survival and apoptosis. Akt is one of the substrates of HSP90, which is crucial for Akt function, forming chaperone–substrate protein complexes 14. The HSP90/Akt pathway was shown to be an important pathway in multiple cell survival and anti‐apoptosis 15, 16. However, it is not clear whether the HSP90/Akt pathway is related to the influence of artemether on the growth and apoptosis of Cal27 cells. In the present study, we found that the HSP90/Akt pathway may be involved in artemether inhibiting Cal27 cell growth and inducing apoptosis.

## Materials and methods

### Cell culture

TSCC cell lines Cal27 and SCC‐15 (BioVector/NTCC, Beijing, China) were maintained in Dulbecco's modified Eagle's medium (DMEM) (4500 mg·L^−1^ glucose) containing 10% fetal bovine serum (Invitrogen, Shanghai, China) and 1% penicillin/streptomycin (Invitrogen). The cells were cultured in incubator containing 5% CO_2_ at 37 °C.

### Preparation of artemether

Artemether (80 mg·mL^−1^) were obtained from Sigma‐Aldrich Co. (St Louis, MO, USA) and dissolved in 80 mL of sterile water to form 10 mg·mL^−1^ storage solution conserved at 4 °C and then diluted to the required concentration with DMEM (4500 mg·L^−1^ glucose, 10% fetal bovine serum, 1% penicillin/streptomycin) when used.

### Cell counting kit‐8 (CCK‐8) assay

Inhibition of cell proliferation was measured by a cell counting kit‐8 (CCK‐8) assay. Cal27 cells and SCC‐15 cells were added into a 96‐well plate at a density of 5.0 × 10^3^ per well and incubated for 24 h under standard conditions. Then, the medium was replaced with DMEM containing various concentrations of artemether (0, 0.025, 0.05, 0.1, 0.15, 0.2 and 0.25 mg·mL^−1^). Cells treated without artemether were used as the control group. After 24 h, 10 μL of CCK‐8 solution was added into each well. After incubation for 3 h, the absorbance at 450 nm was determined by a microplate analyzer, and then the inhibition rate of each group was calculated according to the formula: cell proliferation inhibition rate (%) = (OD_control group _− OD_experimental group_)/OD_control group _× 100%.

### Annexin V‐fluorescein isothiocyanate (FITC)/ propidium iodide (PI) assay

Cal27 cells and SCC‐15 cells were added to a 12‐well plate at a density of 5.0 × 10^3^ per well and incubated with DMEM containing different concentrations of artemether for 24 h and washed twice with phosphate‐buffered saline (PBS). Cells treated without artemether were used as the control group. After centrifugation, the supernatant was discarded, the Cal27 cells and SCC‐15 cells were suspended, and the concentration was adjusted to 1 × 10^6 ^cells·mL^−1^. Next, the cells were stained with 5 μL of Annexin‐V‐FITC solution and 5 μL of PI staining solution and incubated at room temperature in the dark for 15 min, followed by apoptosis as detected by flow cytometry.

### Terminal deoxynucleotidyl transferase dUTP nick end labeling (TUNEL) assay

Cal27 cells and SCC‐15 cells treated with different concentrations of artemether were collected, and then fixed with 4% paraformaldehyde in PBS for 30–60 min at room temperature. After washing with PBS, the cells were re‐suspended in PBS containing 0.1% Triton x‐100 (Sigma‐Aldrich Co.) and incubated in an ice bath for 2 min. Washed twice with PBS, the cells were then incubated with 50 μL of TUNEL reaction mixture (Roche Diagnostics GmbH, Mannheim, Germany) in a humidified chamber and dark room at 37 °C for 1 h. The cells were washed and suspended with PBS, and the apoptotic cells with green fluorescence were detected by fluorescence microscopy.

### Quantitative RT‐PCR assay

Total RNA was extracted from Cal27 cells using TRIzol reagent (Invitrogen, Paisley, UK). The purity and concentration of extracted RNA were determined by the NanoDropTM ND‐1000 (Thermo Fisher Scientific, Waltham, MA, USA). A quantitative RT‐PCR was performed on ABI PRISM 7500 (Applied Biosystems, Foster City, CA, USA) using SYBR Green PCR master mix (TaKaRa, Qingdao, China). The experiment was carried out in triplicate and the expression of RNA was calculated using the 2^−ΔΔCt^ method.

### Western blot analysis

The HSP90α, Akt and β‐actin expression in Cal27 cells were determined by a western blot assay. Total protein was extracted from Cal27 cells using RIPA Lysis Buffer (YEASEN, Shanghai, China; 20101ES60) and protein concentration was determined using a BCA Protein Assay Kit (Pierce, Grand Island, NY, USA). Extracted proteins were separated by electrophoresis on 10% SDS/PAGE and then transferred to a polyvinylidene fluoride membrane (Millipore, Bedford, MA, USA). The membrane was blocked for 1 h with 5% non‐fat milk and then first incubated with the primary antibodies overnight at 4 °C. After washing three times with PBS, the membrane was incubated with secondary antibodies (Santa Cruz Biotechnology, Santa Cruz, CA, USA) at room temperature for 1 h. The exposure bands were scanned and the gray values of the target bands were analyzed using image j, version 1.41 (National Institutes of Health, Bethesda, MD, USA). All the antibodies used in the present study were obtained from Cell Signaling Technology (Danvers, MA, USA).

### Statistical analysis

All values are reported as the mean ± SD. Differences were analyzed with one‐way analysis of variance (ANOVA). *P *< 0.05 was considered statistically significant.

## Results

### Effects of artemether on proliferation and apoptosis in Cal27 cells and SCC‐15 cells

**Figure 1:**
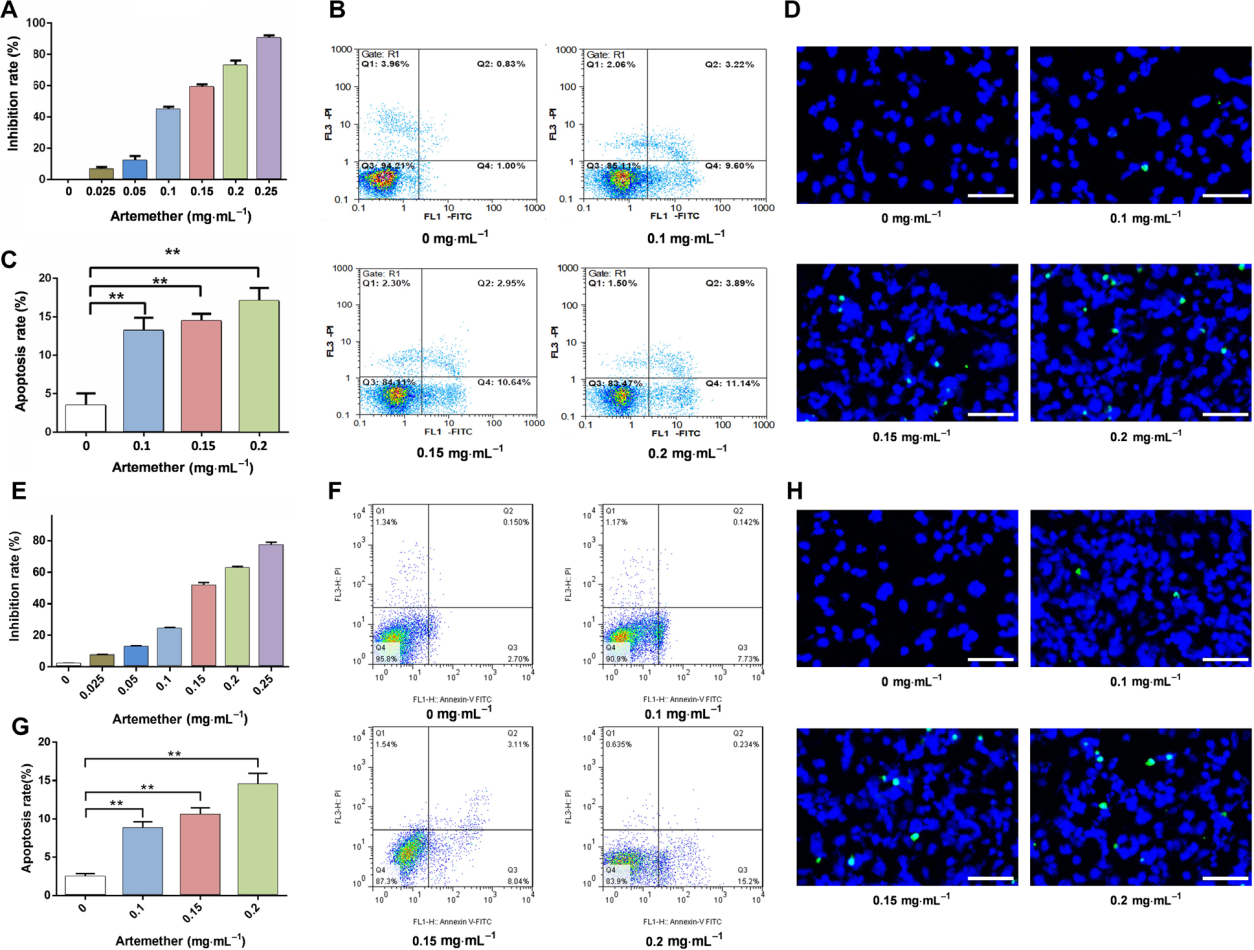
Artemether inhibits proliferation and induces apoptosis in Cal27 cells and SCC‐15 cells. (A, E) Inhibition rate of Cal27 cells and SCC‐15 cells treated without or with artemether (0.025, 0.05, 0.1, 0.15, 0.2 and 0.25 mg·mL^−1^) for 24 h; cell viability was measured by CCK‐8. (B, F) Artemether triggered apoptosis of Cal27 cells and SCC‐15 cells. Annexin V and PI double staining were carried out on Cal27 cells after treatment with artemether for 24 h. (C, G) Effects of different concentrations of artemether on the apoptosis of Cal27 cells and SCC‐15 cells. The error bars represent the SEM of three independent experiments. Differences were analyzed with ANOVA. *P < 0.05; **P < 0.01. (D, H) Apoptosis of Cal27 cells and SCC‐15 cells were detected by TUNEL assay; scale bars = 50 μm.

To confirm the effect of artemether on the growth of TSCC, we treated Cal27 cells and SCC‐15 cells with different concentrations of artemether for 24 h. The CCK‐8 assay showed that the growth of Cal27 cells and SCC‐15 cells was significantly inhibited with an increase in artemether concentration, especially at the concentration of 0.1 mg·mL^−1^ (*P *<* *0.01) (Fig. 1A,E).

Next, we determined whether artemether affected apoptosis in Cal27 cells and SCC‐15 cells. The cells were treated with different concentrations of artemether for 24 h. We then assessed the apoptosis using Annexin V‐FITC and PI staining by flow cytometry. Our results showed that the apoptosis rates of Cal27 cells treated with 0.1, 0.15 and 0.2 mg·mL^−1^ artemether were 13.15 ± 1.01%, 14.44 ± 0.57% and 17.02 ± 1.75%, respectively, and were much higher than the control group (3.45 ± 0.92%, *P *<* *0.01) (Fig. 1B,C). The apoptosis rates of SCC‐15 cells were similar to those of Cal27 cells (*P *<* *0.01) (Fig. 1F,G). In addition, the TUNEL assay was used to further verify the status of cell apoptosis. With an increase in artemether concentration, the number of apoptotic cells with green fluorescence increased, which was consistent with the results of Annexin V FITC/PI assay (Fig. 1D,H). In summary, our results showed that artemether has a strong inhibitory effect on cell proliferation and can induce apoptosis in Cal27 cells and SCC‐15 cells in the first 24 h.

### Effects of artemether on the expression of HSP90 in Cal27 cells

**Figure 2:**
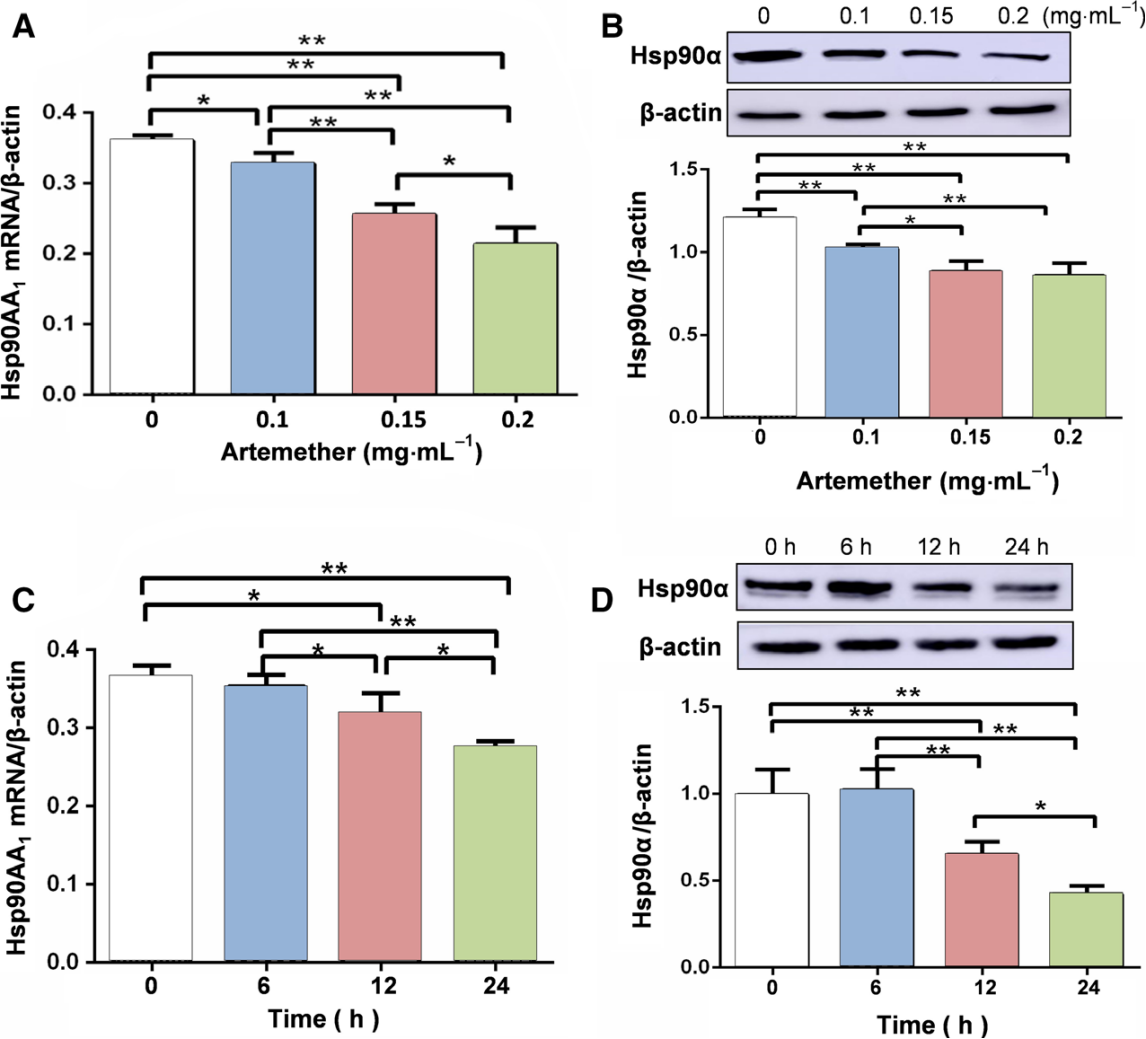
Artemether inhibits the expression of HSP90 in Cal27 cells. (A) Artemether decreased expression level of HSP90AA1 mRNA with an increase in artemether concentration. (B) Western blot analysis of the expression of HSP90α protein treated with different concentrations of artemether. (C) The expression level of HSP90AA1 mRNA in Cal27 cells treated with 0.1 mg·mL^−1^ artemether for different times. (D) The protein expression levels of HSP90α analyzed by western blotting. Cal27 cells treated with 0.1 mg·mL^−1^ artemether for different times. Data are from three independent experiments; error bars indicate the SEM,* n* = 3. Differences were analyzed with ANOVA. *P < 0.05; **P < 0.01.

HSP90 was currently considered as a marker of tumor cells. To investigate the effect of different doses of artemether on the expression of HSP90, the cells were treated with different concentrations of artemether for 12 h. The results showed that the expression of HSP90AA1 mRNA was 0.359 ± 0.009 in the control group and 0.327 ± 0.016, 0.255 ± 0.016 and 0.213 ± 0.024, respectively, in the treatment groups. Compared with the control group, the expression was significantly reduced (*P *<* *0.05) and, with an increase in artemether concentration, the mRNA expression level of HSP90AA1 decreased (Fig. 2A). The protein expression level of HSP90α was 1.20 ± 0.06 in the control group and 1.02 ± 0.03, 0.88 ± 0.06 and 0.86 ± 0.08, respectively, in the treatment groups. By contrasted to the control group, the protein expression of HSP90α was decreased (*P *<* *0.05). Compared with the 0.1 mg·mL^−1^ artemether group, the 0.15 and 0.2 mg·mL^−1^ groups showed lower expression (*P *<* *0.05), although there was no difference between the latter two groups (*P *>* *0.05) (Fig. 2B).

Next, we treated Cal27 cells with 0.1 mg·mL^−1^ artemether for different times. HSP90AA1 mRNA expression in the treatment groups was 0.353 ± 0.014, 0.317 ± 0.27 and 0.227 ± 0.04, respectively. Compared with the control group (0.365 ± 0.015), there was no significant difference in the 6 h treatment group, and expression was significantly reduced after 12 h (*P *<* *0.05) (Fig. 2C). Protein expression of HSP90α was 0.99 ± 0.14 in the control group and 1.02 ± 0.12, 0.65 ± 0.07 and 0.42 ± 0.28, respectively, in the different treatment groups. The expression trend of HSP90α protein was similar to that of HSP90AA1 mRNA (Fig. 2D).

### Effects of artemether on the expression of Akt in Cal27 cells

**Figure 3:**
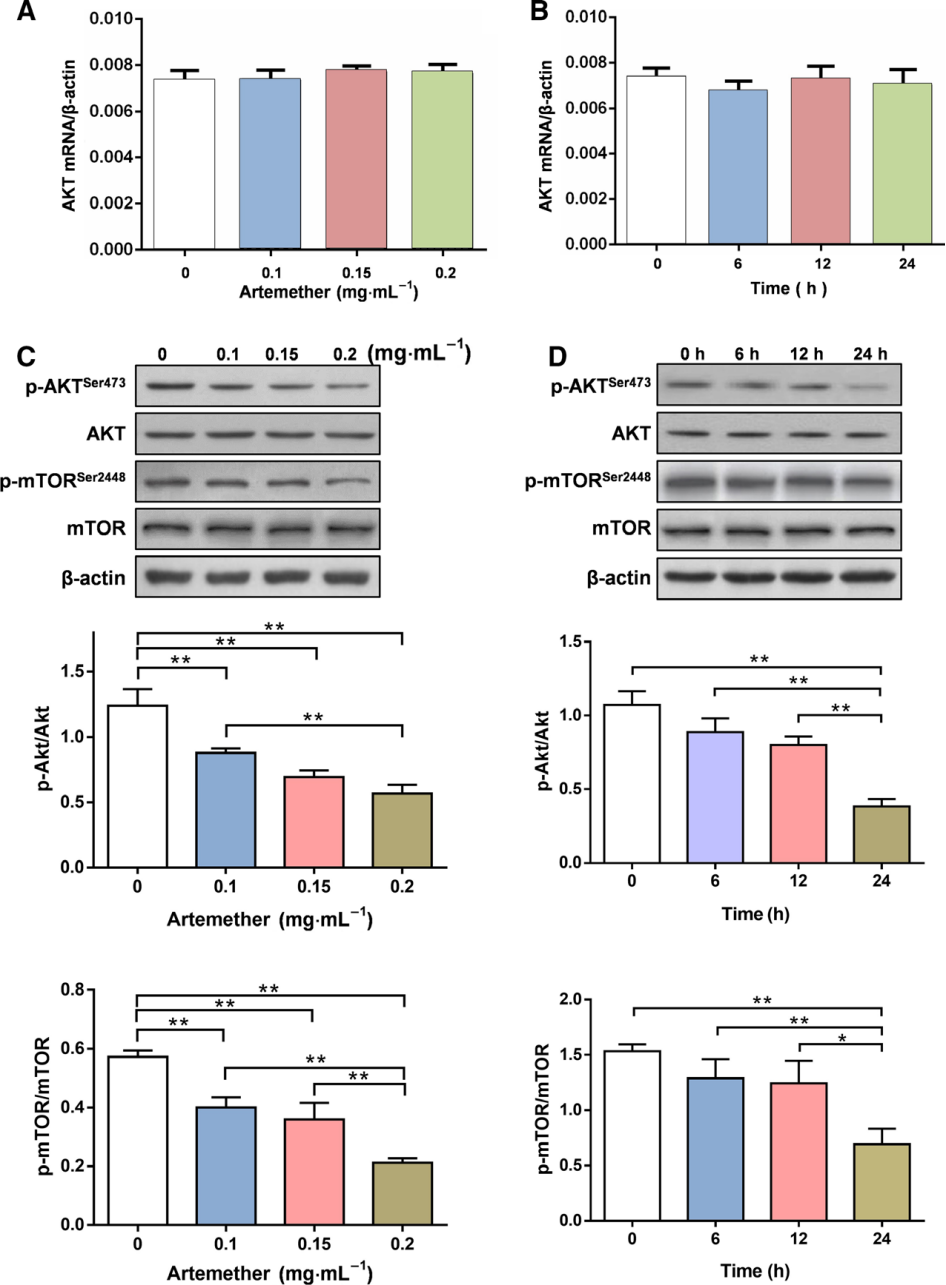
Artemether inhibits the phosphorylation of Akt in Cal27 cells. (A, B) Akt mRNA expressions showed no significant difference in a dose and time‐dependemt manner (C) Western blot analysis of the expression of Akt/p‐Akt and mTOR/p‐mTOR proteins treated with different concentrations of artemether. (D) The protein expression levels of Akt/p‐Akt and mTOR/p‐mTOR analyzed by western blotting. Cal27 cells treated with 0.1 mg·mL^−1^ artemether for different times. Data are from three independent experiments; error bars represent the SEM,* n* = 3. Differences were analyzed with ANOVA. *P < 0.05; **P < 0.01.

Akt, a substrate of HSP90, is tightly connected with signaling pathway induced by growth factor. In addition, a previous study has indicated that the Akt/mammalian target of rapamycin (mTOR) pathway is involved in regulating the proliferation and apoptosis of cancer cells 17. Accordingly, we investigated whether the HSP90/Akt/mTOR axis is involved in the apoptosis of Cal27 cells. The data showed that there were no significant changes in Akt mRNA expression compared to the control group in a dose and time‐dependemt manner (*P *>* *0.05) (Fig. 3A,B). After 24 h of treatment with different concentrations of artemether, expression of Akt and mTOR showed no significant change compared to the control group (*P *>* *0.05), although the expression of p‐Akt and p‐mTOR was significantly lower compared to the control group (*P *<* *0.01) and there were significant differences among the treatment groups (*P *<* *0.01) (Fig. 3C). After treatment with artemether at 0.1 mg·mL^−1^ for different times, Akt and mTOR expression showed no significant changes compared to the control group (*P *>* *0.05), although the expression of p‐Akt and p‐mTOR was significantly decreased with treatment time (*P *<* *0.01) (Fig. 3D).

## Discussion

Artemether, a derivative extracted from a Chinese traditional herb, has been widely prescribed in patients diagnosed with malaria 18. In recent years, emerging evidence has demonstrated the potential therapeutic effects of artemether in several malignancies, such as glioma, liver cancer and breast cancer 19. In the present study, we found that artemether decreased the expression levels of HSP90, p‐Akt and p‐mTOR in a dose and time‐dependemt manner, suggesting that the HSP90/Akt/mTOR axis is related to artemether‐induced apoptosis in Cal27 cells.

TSCC is the most common type of oral squamous cell carcinoma, with strong invasiveness, easy occurrence of neck lymphatic metastasis and poor outcomes 20. The quality of life in TSCC patients is often reduced by dysphasia, as well as difficulties in chewing and swallowing. Surgical resection, radiotherapy and chemotherapy have yielded significant advancements, although the death and recurrence rates of this cancer are still high 21, 22. Accumulating evidence shows that artemisinin and its derivatives have obvious cytotoxic and inhibitory effects on different cancer cells. For example, artemisinin can inhibit inflammatory response via regulating nuclear factor‐kappa B and mitogen‐activated protein kinase signaling pathways 23. Dihydroartemisinin, a semi‐synthetic derivative of artemisinin, results in significant inhibition of human hepatocellular carcinoma both *in vitro* and *in vivo* via the induction of mitochondria‐dependent apoptosis 24. By inhibiting the expression of cell cycle genes and c‐Myc, artemether promoted caspase‐3 and PARP1 cleavage, and significantly inhibited the proliferation and induced apoptosis of diffuse large B cells 8. Another study found that artemether (> 300 μmol·L^−1^) significantly reduced the proliferation of neuroblastoma cell lines, including SH‐SY5Y, SK‐N‐SH and SK‐NBE2. Moreover, the cell viability and DNA synthesis in tumor cells were remarkably lower in the presence of artemether and doxorubicin than in doxorubicin‐treated cells alone 25. We found that artemether (> 0.1 mg·mL^−1^) significantly inhibited the proliferation of Cal27 cells, and induced cell apoptosis in the first 24 h.

HSP90 is a molecular chaperone that affects cell proliferation or apoptosis via interaction with ‘chaperone proteins’ involved in transcriptional regulation and signal transduction pathways. Most of its chaperone proteins are proteins that control cell differentiation and inhibit cell apoptosis. Akt is a chaperone protein of HSP90, and inhibition of HSP90 induces Akt degradation, thereby inhibiting cancer cell proliferation and inducing apoptosis 26. The HSP90/Akt pathway is an important pathway in the cells and environments, because HSP90/Akt complex lysis is crucial for HSP90/Akt complex instability and triggering of the apoptosis signal 27, 28, 29. Li *et al*. observed that myocardial calpain induces caspase‐3 activation and apoptosis. The potential mechanism include decreased HSP90/p‐Akt protein levels induced by myocardial calpain and inhibition of Akt signaling, which increases caspase‐3 activity and apoptosis during sepsis 15. Ke *et al*. 16 demonstrated that exogenous H_2_S can prevent cytotoxicity, apoptosis and excessive production of reactive oxygen species, as well as decrease superoxide dismutase activity and metalloproteinase dissipation, by activating the HSP90/Akt pathway, so as to protect H9c2 myocardial cells from mercury‐induced damage. In addition, some studies have shown that some HSP90 inhibitors can induce autophagy via inactivation of the Akt/mTOR pathway 30, 31, 32, 33. Another study has also indicated that the Akt/mTOR pathway is involved in regulating the proliferation and apoptosis of cancer cells 17. In summary, we have investigated whether the HSP90/Akt/mTOR axis is involved in apoptosis of Cal27 cells. Our data show that the artemether inhibited growth and induced apoptosis in Cal27 cells and also that the the expression of HSP90, p‐Akt and p‐mTOR is significantly down‐regulated. The potential mechanism may be that artemether inhibits the expression of HSP90, reducing the activity of Akt by inhibiting the phosphorylation of Akt, then reducing the phosphorylation of mTOR and, finally, inducing apoptosis of Cal27 cells. However, whether there is a direct relationship between HSP90 and Akt/mTOR is not confirmed in the present study and this requires further investigation.

## Conclusions

We demonstrate that artemether can inhibit the growth and induce apoptosis of Cal27 cells and also that its mechanism may be related to HSP90/Akt pathway. The present study provides a potential novel therapy for TSCC.

## Conflict of interest

The authors declare no conflict of interest.

## Author contributions

YWH conceived and designed the project. JHW and LL acquired the data. YTW and XBR analyzed and interpreted the data. KL wrote the paper. All authors have read and concurred with the final manuscript submitted for publication.
